# Supplementary material for: Modulation of Salmonella virulence by a novel SPI-2 injectisome effector that interacts with the dystrophin-associated protein complex
Source: mBio. 2024 Jun 21;15(7):e01128-24. doi: 10.1128/mbio.01128-24 (PMC11253597; doi:10.1128/mbio.01128-24)
Supplement: Supplemental figures and tables — Figures S1-S6 and Tables S1-S3. [file mbio.01128-24-s0001.docx]

**Supplementary Information for Yu et al.,**


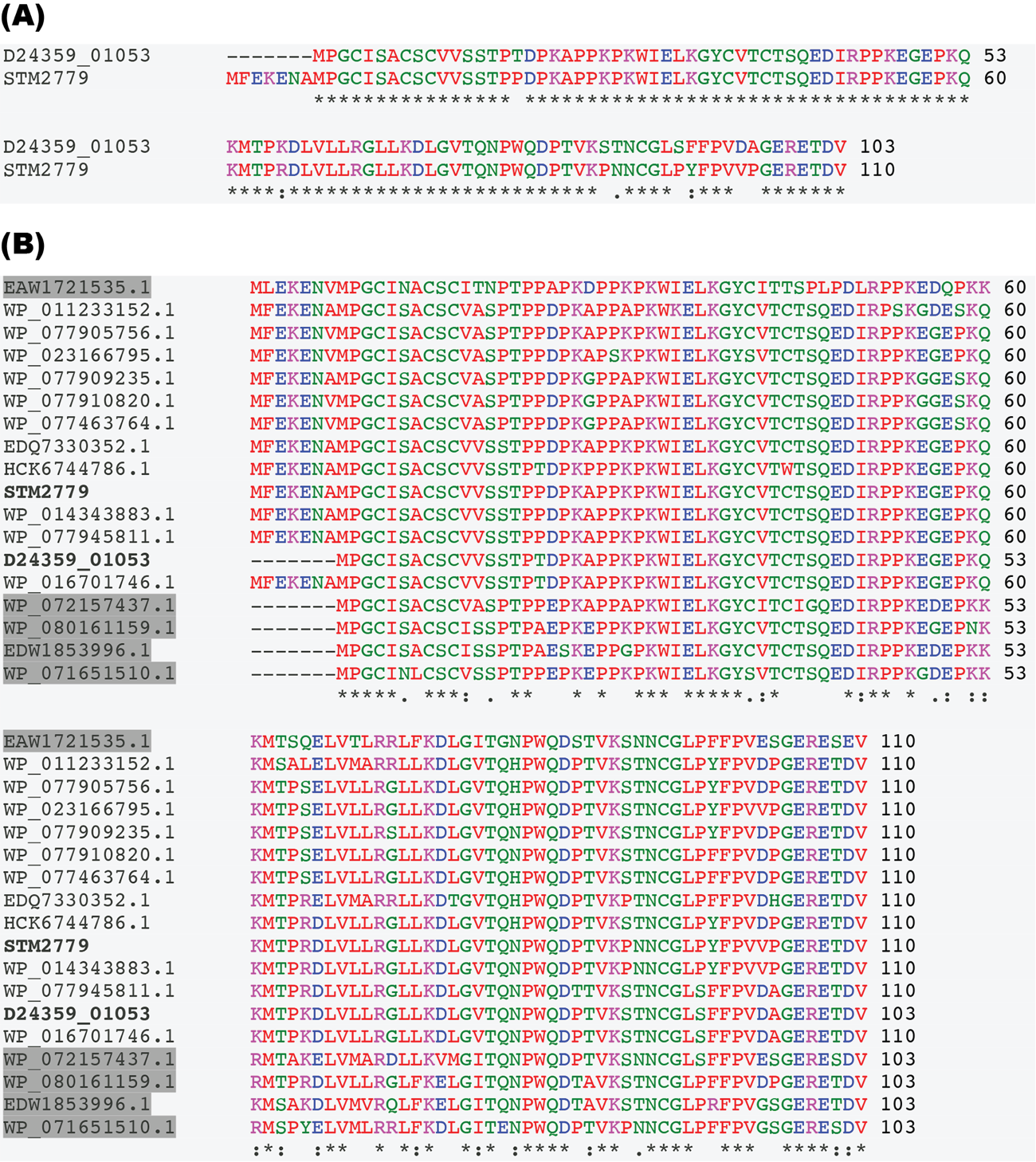


**sFig.1 Alignment of SseM from different subspecies and different serovars of *Salmonella* *enterica*.** (A) Alignment of D24359_01053 and STM2779. Identities: 95/103=92%; positives: 97/103=94%. (B) Alignment of SseM protein sequences. Accession numbers for non-*enterica* subspecies are highlighted in grey. EAW1721535.1: *S. enterica* subsp. *indica*; WP_080161159.1: *S. enterica* subsp. *arizonae*; WP_072157437.1: *S. enterica* subsp. *salamae*; EDW1853996.1: *S. enterica* subsp. *diarizonae*; WP_071651510.1: *S. enterica* subsp. *houtenae*; WP_011233152.1: serovar Paratyphi A; WP_077905756.1: serovars Agona, Indiana, Goldcoast, Brancaster, Senftenberg; WP_023166795.1: serovars Kentucky, Senftenberg and Gallinarum; WP_077909235.1: serovars Muenchen, Litchfield, Manhattan; WP_077910820.1: serovars Paratyphi B, Java; WP_077463764.1: serovars Heidelberg, Newport, Infantis, Hadar etc. EDQ7330352.1: serovar Paratyphi C; HCK6744786.1: serovars Anatum, Eko, Typhi; WP_014343883.1: serovars Typhimurium, Saintpaul, Paratyphi B, Berta, Bareilly; WP_077945811.1: serovars Brunei, Newport; WP_016701746.1: serovar Enteritidis.


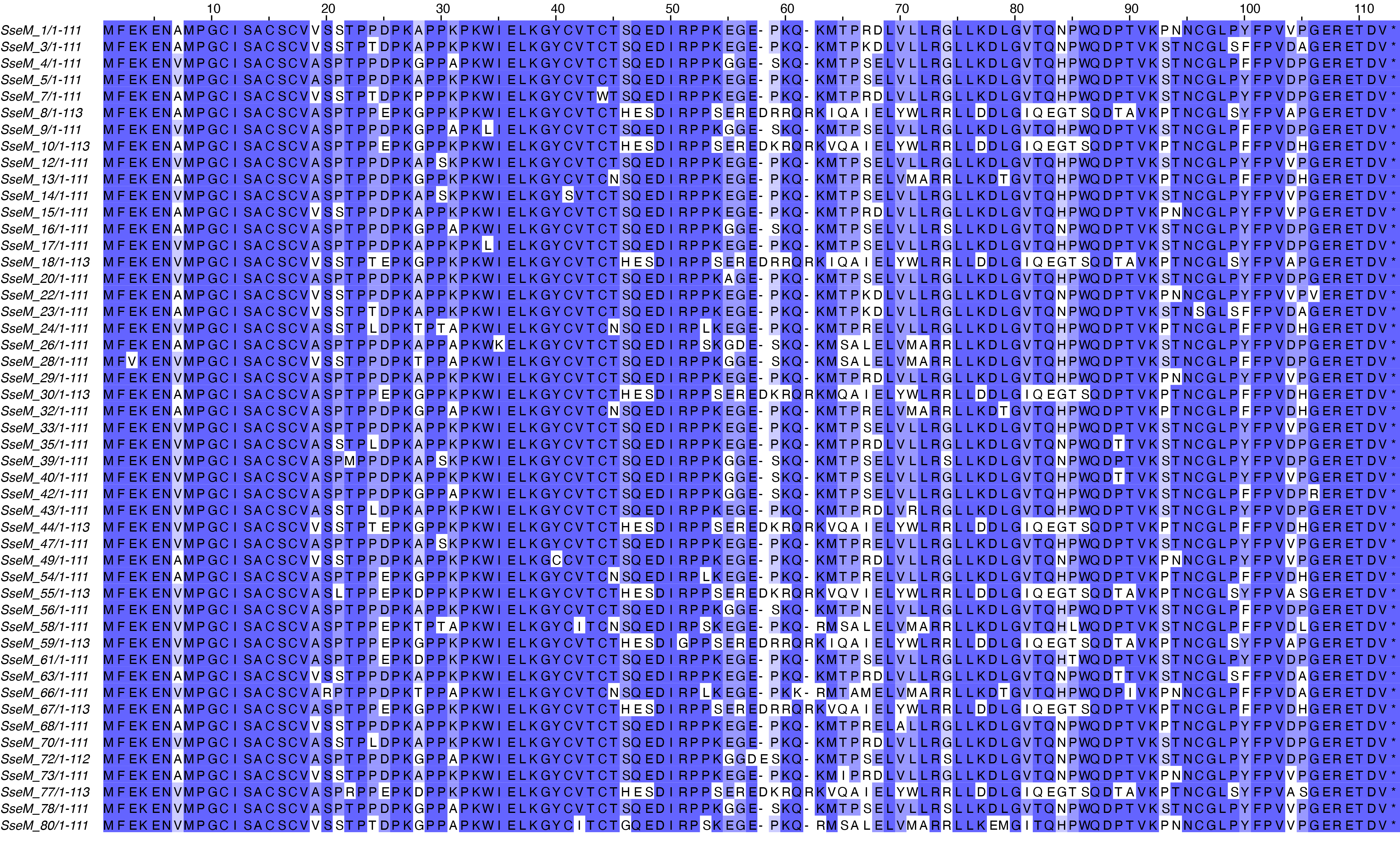


**sFig.2 Alignment of SseM variants**

Sequence alignment of SseM variants from Fig. 1D.


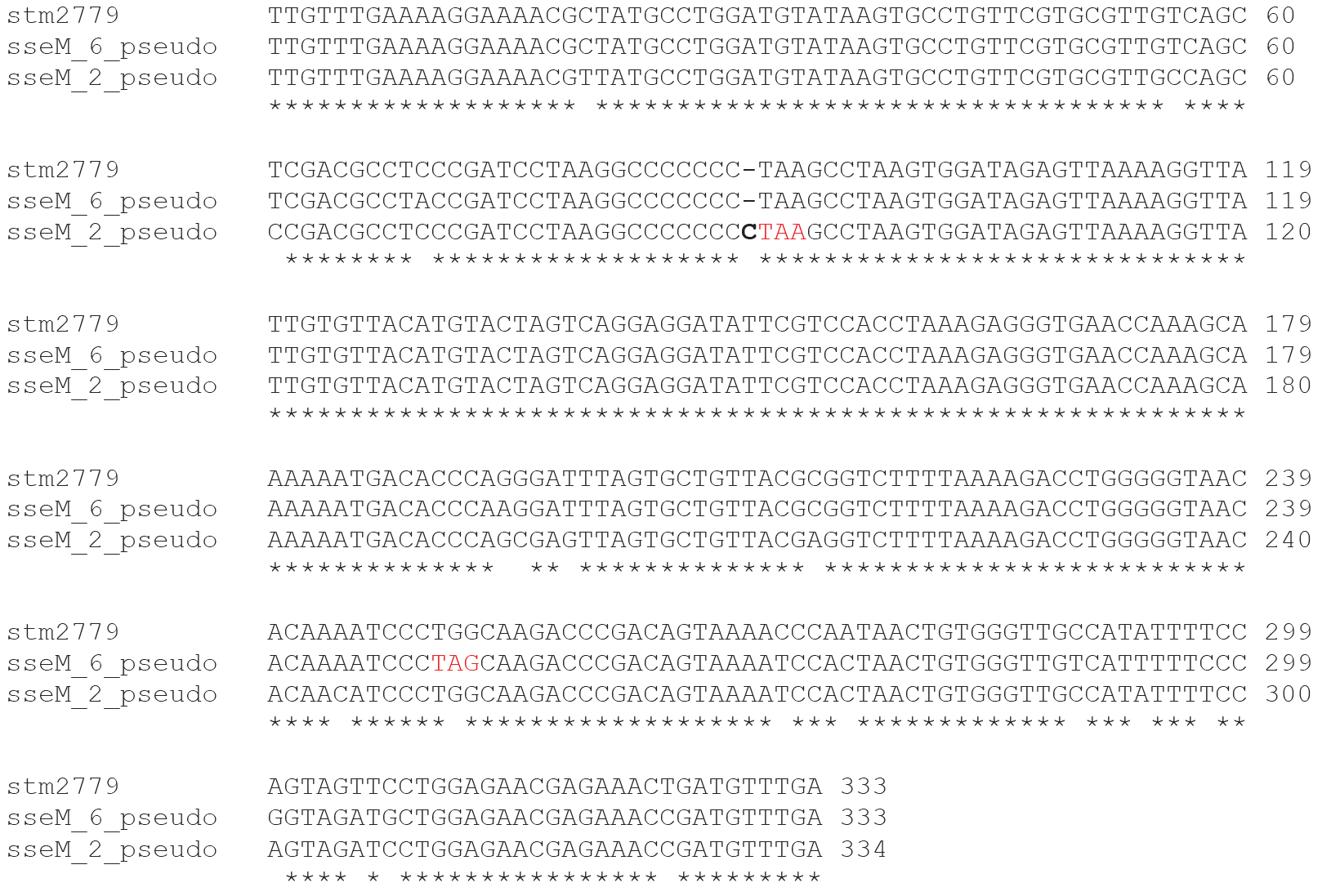


**sFig.3 Alignment of *stm2779*, *sseM_2_pseudo* and *sseM_6_pseudo*.** The extra ‘C’ (underlined bold font) in *sseM_2_pseudo* results in a stop codon (red fonts) after the predicated 30^th^ amino acid. The mutation of the predicated 84^th^ codon TGG to TAG (red font) in *sseM_6_pseudo* results in a truncated protein missing the last 28 residues of SseM.

**
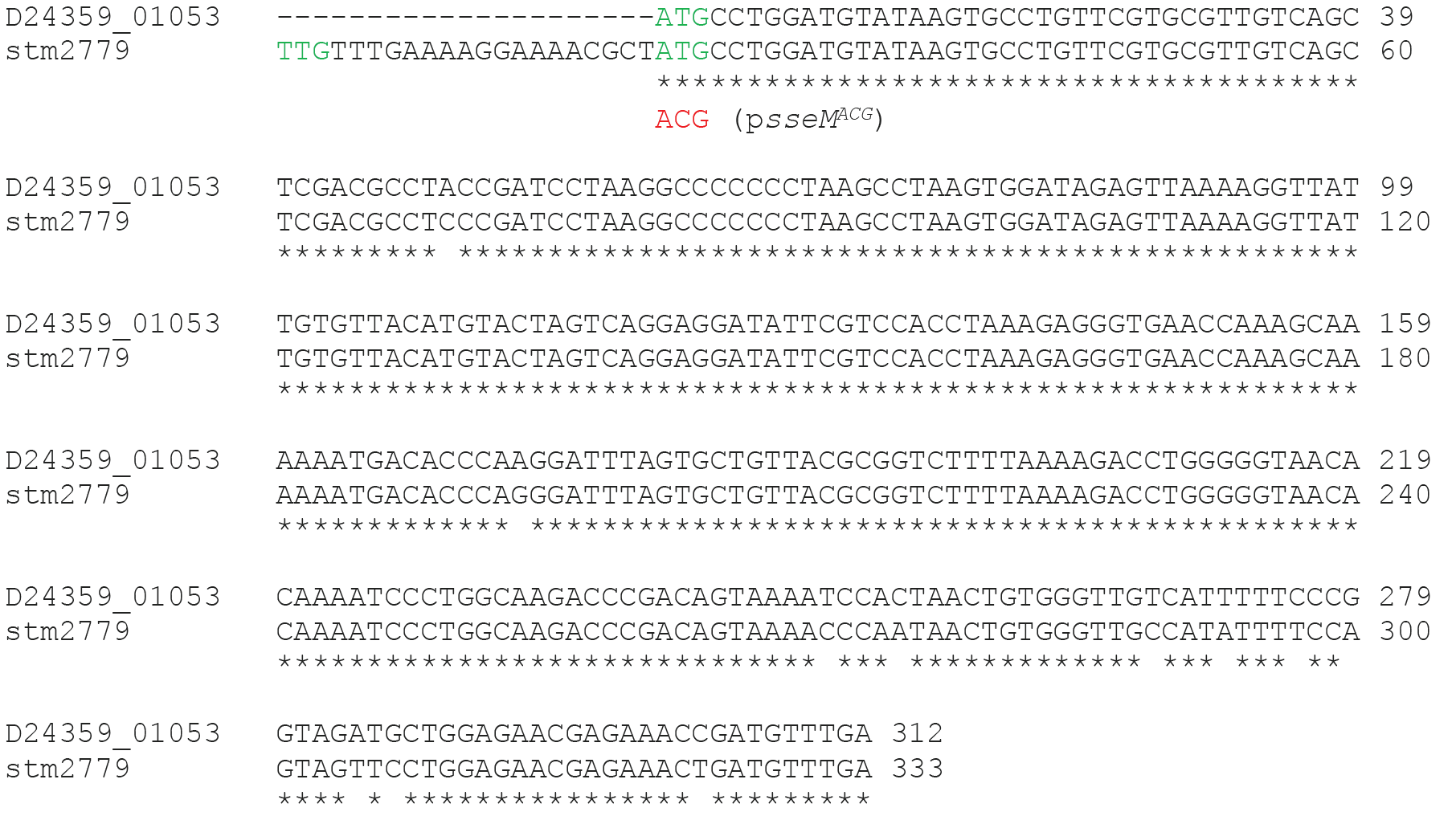
**

**sFig.4** **Alignment of *stm2779* and *D24359_01053*.** Green fonts indicate the predicated start codon (TTG) and actual start codon (ATG) of *stm2779*. Plasmid p*sseM^ACG^* was constructed by changing the ATG (green fonts) to ACG (red fonts) on p*sseM*.

**
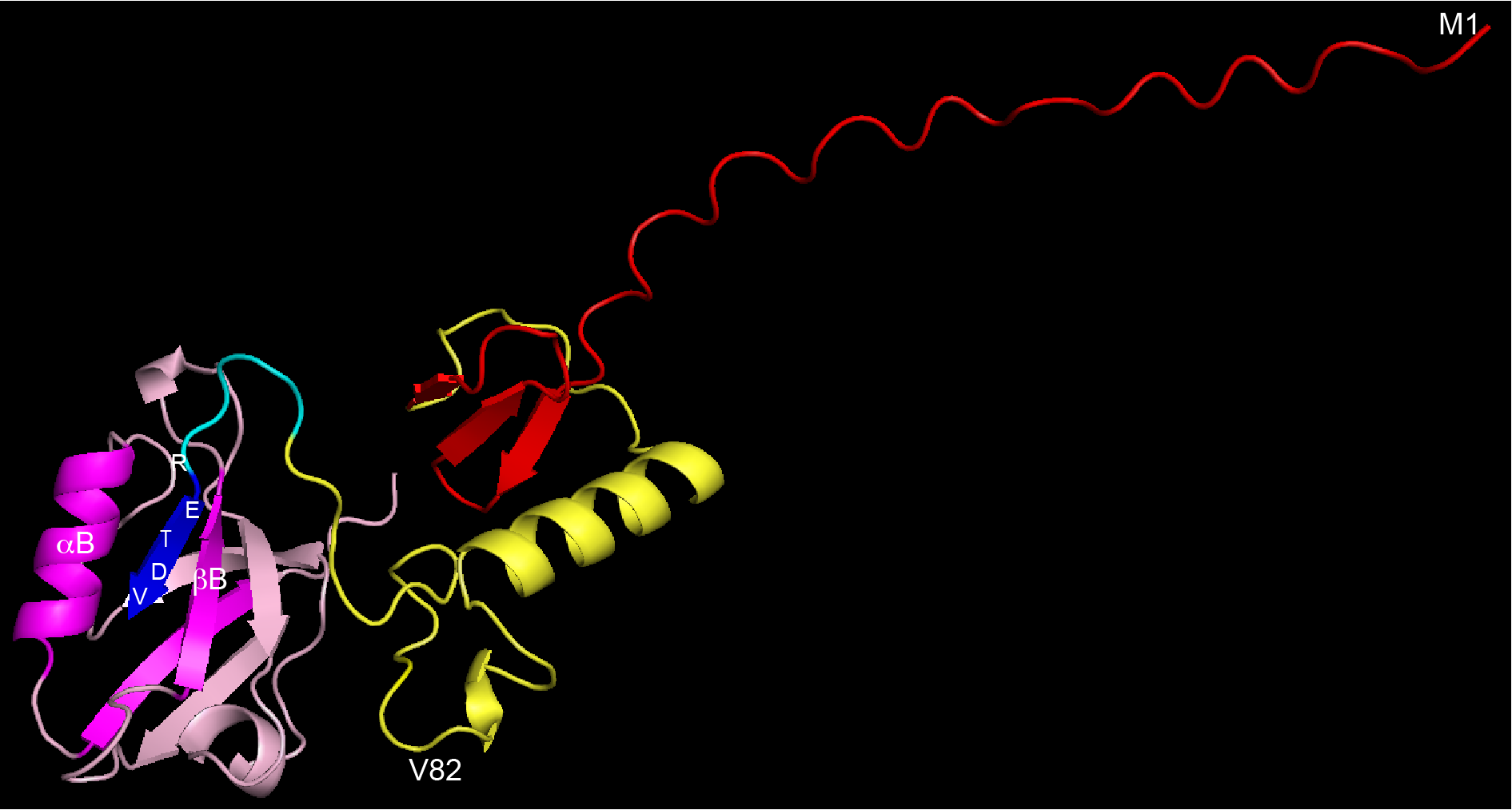
**

**sFig.5** **Model of complex between SseM and β-2-syntrophin PDZ domain (coloured in magenta and light pink) predicted with AlphFold Colab Multimer.** The different colours of SseM represent the model confidence: blue (very high, pLDDT >90), cyan (confident, 90 > pLDDT > 70), yellow (low, 70 > pLDDT > 50), and red (very low, pLDDT < 50). The M1, V82 and RETDV motif of SseM are annotated. The model confidence of β-2-syntrophin PDZ domain are indicated in different colours: magenta (very high, pLDDT > 90) and light pink (confident, 90 > pLDDT > 70).

**
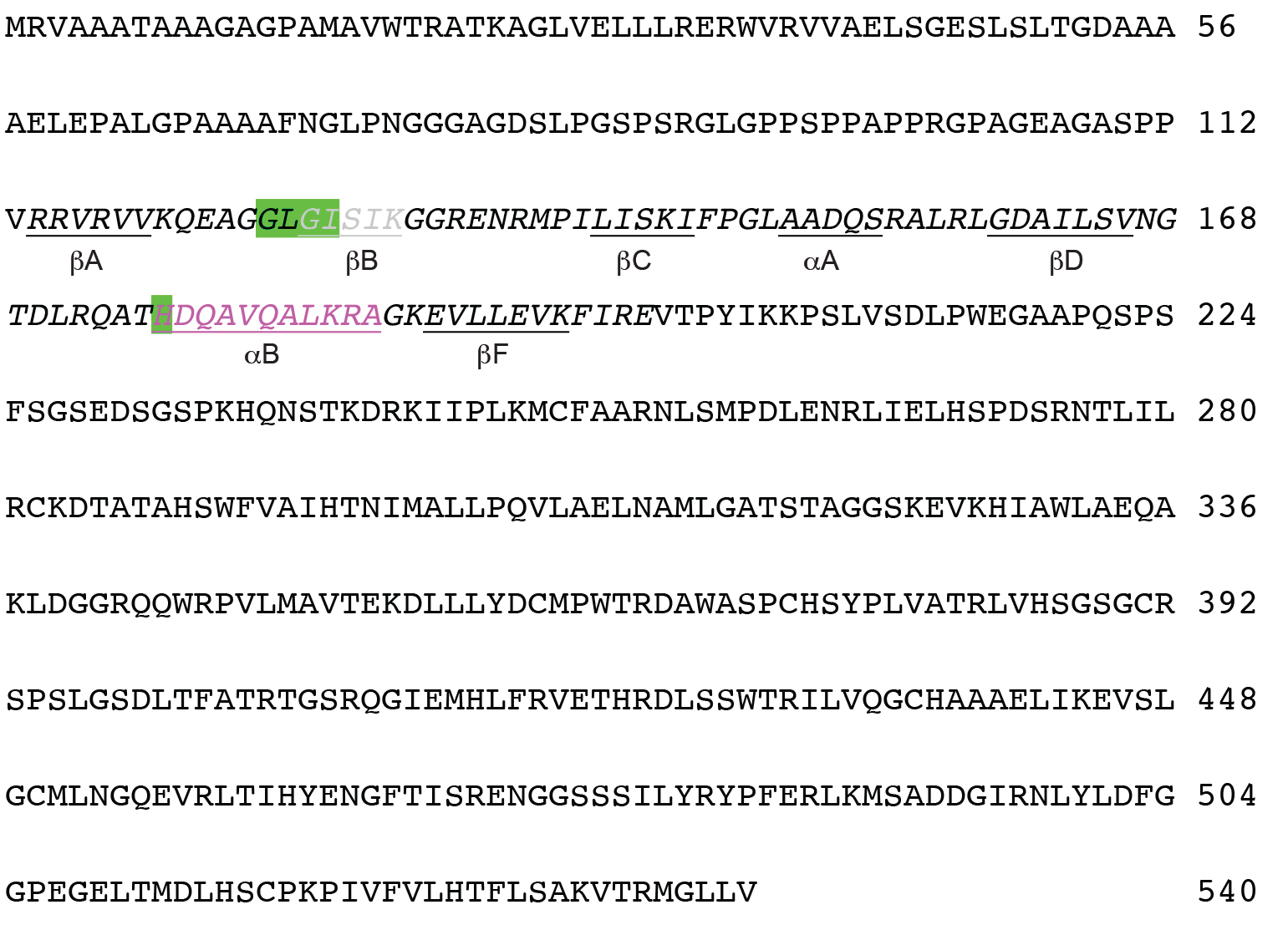
**

**sFig.6** **Predicted secondary structural features of β-2-syntrophin PDZ domain with AlphFold Colab Multimer.** The secondary structure of PDZ domain (italic fonts) are indicated. Mutated residues of PDZ domain of β-2-syntrophin used in Fig. 3F are highlighted in green.

**Supplemental Table 1: *Salmonella* strains and plasmids used in this work**

| **Strain or Plasmid** | **Description** | **Reference or source** |
| --- | --- | --- |
| ***S.* Enteritidis** |  |  |
| D24359 | Wild-type | [25] |
| *ΔspiC* | *ΔspiC::Km* in *S.* Enteritidis D24359 | This work |
| *ΔspiCssaC* | *ΔspiCssaC::Km* in *S.* Enteritidis D24359 | This work |
| ***S.* Typhimurium** |  |  |
| 12023 | Wild-type | NCTC |
| *ΔsseM* | *ΔsseM* in 12023 | This work |
| *ΔpipB2* | *ΔpipB2* in 12023 | This work |
| *ΔpipB2^promoter^* | *ΔpipB2^promoter^* in 12023 | This work |
| HH109 | *ΔssaV::aphT* in 12023 | [2] |
| P3F4 | *ssrA::mTn5* in 12023 | [12] |
| *ΔsteC* | *ΔsteC* in 12023 | [50] |
| wt::Km | *Δstm0587::Km* in 12023 | Lab stock |
| **Plasmids** |  |  |
| pWSK29 | Low copy number cloning vector, Ap^r^ | [51] |
| p*ssaGpr* | *ssaGpromoter* inserted in pWSK29, Ap^r^ | [6] |
| p*sseM* | *sseM* in p*ssaGpr*, Ap^r^ | This work |
| p*sseM^ACG^* | *sseM^ACG^* in p*ssaGpr*, Ap^r^ | This work |
| p*sseM-HA* | *sseM-HA* in p*ssaGpr*, Ap^r^ | This work |
| p*sseM^V103A^* | *sseM^V103A^* in p*ssaGpr*, Ap^r^ | This work |
| p*sseM^SEN^* | *sseM* of *S.* Enteritidis D24359 in p*ssaGpr*, Ap^r^ | This work |
| M6pblast-GFP | Transfection vector, Ap^r^ | [52] |
| pCG36 | Transfection plasmid expressing Flag-SteD, Km^r^ | C. Godlee |
| pCG189 | Transfection plasmid expressing mCherry-SteD, Ap^r^ | C. Godlee |
| p*gfp::spvD* | *spvD* inserted in M6pblast-GFP, Ap^r^ | This work |
| p*gfp::sseM* | *sseM* inserted in M6pblast-GFP, Ap^r^ | This work |
| p*gfp::sseM-HA* | *sseM-HA* inserted in M6pblast-GFP, Ap^r^ | This work |
| p*gfp::sseM^RE2A^* | *sseM^RE2A^* inserted in M6pblast-GFP, Ap^r^ | This work |
| p*gfp::sseM^T101A^* | *sseM^T101A^* inserted in M6pblast-GFP, Ap^r^ | This work |
| p*gfp::sseM^V103A^* | *sseM^V103A^* inserted in M6pblast-GFP, Ap^r^ | This work |
| p*gfp::sseM^SEN^* | *sseM* of D24359 inserted in M6pblast-GFP, Ap^r^ | This work |
| p*flag::sseM* | *sseM* replaced steD of pCG36, Km^r^ | This work |
| p*flag::spvD* | *spvD* replaced steD of pCG36, Km^r^ | This work |
| p*mCherry::sseM* | *sseM* replaced steD of pCG189, Ap^r^ | This work |
| p*mCherry::sseM^V103A^* | *sseM^V103A^* replaced steD of pCG189, Ap^r^ | This work |
| p*gfp::SNTB2* | *SNTB2* inserted in M6pblast-GFP, Ap^r^ | This work |
| p*gfp::SNTB2^GLGI4A^* | *SNTB2^GLGI4A^* inserted in M6pblast-GFP, Ap^r^ | This work |
| p*gfp::SNTB2^H176Y^* | *SNTB2^H176Y^* inserted in M6pblast-GFP, Ap^r^ | This work |
| p*gfp::SNTB2^H176V^* | *SNTB2^H176V^* inserted in M6pblast-GFP, Ap^r^ | This work |
| pX330 | Transfection vector for making knockout cell line, Ap^r^ | [59] |
| p361 | gRNA targeting 871-891 nt of SNTB2 on pX330, Ap^r^ | This work |
| p363 | gRNA targeting 1072-1092 nt of SNTB2 on pX330, Ap^r^ | This work |

**Supplemental Table 2: Primers used in this work.**

| Name | Nucleotide sequence (5’ to 3’) | Use of primer |
| --- | --- | --- |
| spiCd1 | TAGAAACTCCCATTTATGTCTGAGGAGGGATTCATGCTGGCAGTGTA  GGCTGGAGCTGCTTC |  |
| spiCd2 | CGTTTATTTACTACCATTTTATACCCCACCCGAATAAAGTTTCATATG  AATATCCTCCTTAGT | With spiCd1 to make Δ*spiC::Km* mutant. |
| ssaCd2 | TGGATTTGTTTACCTTGTTTAAACCCTCCCAGCAATAGACTTTGCCA  TATGAATATCCTCCTTAGT | With spiCd1 to make Δ*spiCssaC::Km* mutant. |
| XJY222 | ATGCCTGGATGTATAAGTGCCTGTTCGTGCGTTGTCAGCTCGACGC  GTGTAGGCTGGAGCTGCTTC |  |
| XJY223 | TTACTGTCGGGTCTTGCCAGGGATTTTGTGTTACCCCCAGGTCTTCA  TATGAATATCCTCCTTAGT | With XJY222 to make Δ*sseM::Km* mutant. |
| XJY257 | TTTGGCTGCTATTGTGTAAGCCAGACAGCAACGCGTCGTGGTGTAG  GCTGGAGCTGCTTC |  |
| XJY258 | ACCAGCCAGACTATCGAGTGAACGCTCCATATATTTTCTCCATATGA  ATATCCTCCTTAGT | With XJY257 to make Δ*pipB2^promoter^::Km*  mutant. |
| XJY401 | ATGGAGCGTTCACTCGATAGTCTGGCTGGTATGGCTAAATCTGCGT  GTAGGCTGGAGCTGCTTC |  |
| XJY402 | AATATTTTCACTATAAAATTCGTTAAAGAGTGTTTGTGTGCTTGCATA  TGAATATCCTCCTTAGT | With XJY401 to make Δ*pipB2::Km* mutant. |
| XJY238 | TCAAGCTTACTAAAGCGAATTGTTTGAAAAGGAAAACGCTATGCCTG  GATG | With XJY239 to construct p*sseM*. |
| XJY239 | AACTGCAGTCAAACATCAGTTTCTCGTTCTCCAGGAAC |  |
| XJY240 | TCAAGCTTACTAAAGCGAATTGTTTGAAAAGGAAAACGCTACGCCTG  GATGTATAAGTG CCTGTTC | With XJY239 to construct p*sseM^ACG^*. |
| XJY241 | AACTGCAGTCAAGCATAATCTGGAACATCATATGGATAAACATCAGTT  TCTCGTTCTCCAGGAAC | With XJY238 to construct p*sseM-HA.* |
| XJY337 | ATAAGAATGCGGCCGCCTGCAGTCAAACATCGGTTTCTCGTTCTCCA  GC | With XJY238 to construct p*sseM^SEN^*,  With XJY253 to construct p*gfp*::*sseM^SEN^*. |
| XJY338 | ATAAGAATGCGGCCGCCTGCAGTCAGGCATCAGTTTCTCGTTCTCCA  GGAACTACTGGAAAATATG | With XJY238 to construct p*sseM^V103A^*,  With XJY253 to construct p*gfp*::*sseM^V103A^*  and p*mCherry*::*sseM^V103A^*. |
| XJY284 | CATGCCATGGGTGGCGGAGGGTCGGGTGGAGGAGGCAGTAGAGTTT  CTGGTAGTGCGTCATCCC |  |
| XJY285 | ATAAGAATGCGGCCGCTCAATCGTGTTTTTCATCATAAGCCCTG | With XJY284 to construct p*gfp*::*spvD*  and p*flag*::*spvD*. |
| XJY253 | CATGCCATGGGTGGCGGAGGGTCGGGTGGAGGAGGCAGTCCTGGAT  GTATAAGTGCCTGTTCGTG |  |
| XJY254 | ATAAGAATGCGGCCGCTCAAACATCAGTTTCTCGTTCTCCAGG | With XJY253 to construct p*gfp*::*sseM*,  p*flag*::*sseM* and p*mCherry*::*sseM*. |
| XJY325 | ATAAGAATGCGGCCGCTCAAGCATAATCTGGAACATCATATGGATAAA  CATCAGTTTCTCGTTCTCCAGGAAC | With XJY253 to construct p*gfp*::*sseM-HA*. |
| XJY329 | ATAAGAATGCGGCCGCCTGCAGTCAAACATCAGTGGCAGCTTCTCCA  GGAACTACTGGAAAATATG | With XJY253 to construct p*gfp*::*sseM^RE2A^*. |
| XJY336 | ATAAGAATGCGGCCGCCTGCAGTCA AACATCCGCTTCTCGTTCTCCA  GGAACTACTGGAAAATATG | With XJY253 to construct p*gfp*::*sseM^T101A^*. |
| XJY361 | CACCGGGTGTGGATAGCTACGAACC | With XJY362 to construct p*361*. |
| XJY362 | AAACGGTTCGTAGCTATCCACACCC |  |
| XJY363 | CACCGTGCTCTATGACTGTATGCCG | With XJY364 to construct p*363*. |
| XJY364 | AAACCGGCATACAGTCATAGAGCAC |  |
| XJY377 | CATGACATGTGTGGCGGAGGGTCGGGTGGAGGAGGCAG |  |
| XJY378 | CTTGATGCTCGCGGCTGCAGCGCCCGCCTCTTGCTTCACCACCCGC |  |
| XJY379 | AAGAGGCGGGCGCTGCAGCCGCGAGCATCAAGGGCGGGCGCGAGAA  CCGG |  |
| XJY380 | ATAAGAATGCGGCCGCTCATACAAGCAGTCCCATACGAGTGAC | With XJY377/378/379 to  construct p*gfp::SNTB2^GLGI4A^.* |
| XJY392 | CTGCACGGCCTGGTCGTaGGTGGCCTGGCGCAGGTCGGTGCCGT |  |
| XJY393 | ACCGACCTGCGCCAGGCCACCtACGACCAGGCCGTGCAGGCGCTGAA  GC | With XJY377/378/392 to  construct p*gfp::SNTB2^H176Y^.* |
| XJY394 | CCTGCACGGCCTGGTCGacGGTGGCCTGGCGCAGGTCGGTGCCGT |  |
| XJY395 | ACCGACCTGCGCCAGGCCACCgtCGACCAGGCCGTGCAGGCGCTGAA  GCG | With XJY377/378/394 to  construct p*gfp::SNTB2^H176V^.* |

**Supplemental Table 3: Antibodies used in this work.**

| Antibodies | Source | Use (dilution) |
| --- | --- | --- |
| Rabbit anti-SseM | Produced by GenScript | IB (1:2,000) |
| Mouse anti-DnaK (8E2/2) | Enzo | IB (1:10,000) |
| Rabbit anti-SseB | Lab stock | IB (1:10,000) |
| Rabbit anti-GAPDH (ab9485) | Abcam | IB (1:2,000) |
| Rat anti-GFP (3H9) | Chromotek | IB (1:2,500) |
| Mouse anti-α-dystrobrevin (sc-271630) | Santa Cruz | IB (1:1,000) |
| Mouse anti-syntrophin (clone 1351) | BioLegend | IB (1:1,000) |
| HRP-conjugated goat anti-rabbit Igs (P0448) | Agilent (Dako) | IB (1:5,000) |
| HRP-conjugated goat anti-mouse Igs (P0447) | Agilent (Dako) | IB (1:5,000) |
| HRP-conjugated goat anti-rat IgG (#7077) | Cell Signalling | IB (1:2,000) |
| Mouse anti-HA (HA.11) | BioLegend | IF (1:500) |
| Goat anti-CSA-1 | Kirkegaard and Perry Laboratories | IF (1:200) |
| Alexa Fluor 488-conjugated donkey anti-mouse IgG | Thermo Fisher Scientific | IF (1:400) |
| Alexa Fluor 555-conjugated donkey anti-goat IgG | Thermo Fisher Scientific | IF (1:400) |

IB: immunoblotting; IF: immunofluorescence labelling.
